# Efficacy and safety of teriparatide vs. bisphosphonates and denosumab vs. bisphosphonates in osteoporosis not previously treated with bisphosphonates: a systematic review and meta-analysis of randomized controlled trials

**DOI:** 10.1007/s11657-024-01447-7

**Published:** 2024-09-23

**Authors:** Mingnian Li, Zhuoqi Ge, Benqi Zhang, Li Sun, Zhongyuan Wang, Tao Zou, Qi Chen

**Affiliations:** 1 College of Pharmacy, Guizhou Medical University, Guiyang, Guizhou China; 2 Department of Pharmacy, Guizhou Provincial People’s Hospital, Guiyang, Guizhou China; 3 Department of Pharmacy, Beijing Jishuitan Hospital Guizhou Hospital, Guiyang, Guizhou China

**Keywords:** Teriparatide, Denosumab, Bisphosphonates, Osteoporosis, Randomized controlled trials

## Abstract

***Summary*:**

The study found that in osteoporosis patients who had not previously received bisphosphonate treatment and were in a treatment cycle of over 12 months, both teriparatide and denosumab significantly increased bone mineral density compared to bisphosphonates. Additionally, teriparatide was also shown to significantly decrease the risk of fractures.

**Objective:**

The systematic review and meta-analysis aimed to assess and compare the safety and efficacy of teriparatide vs. bisphosphonates and denosumab vs. bisphosphonates in patients with osteoporosis who had not previously received bisphosphonates.

**Methods:**

We conducted a search of published literature from inception to May 31, 2023, including databases such as PubMed, Embase, Cochrane Library, CNKI, SinoMed, VIP, and WanFang. The study only included head-to-head randomized controlled trials (RCTs) that compared teriparatide and denosumab with bisphosphonates to treat patients with osteoporosis. Fixed-effect model and random-effect model were used due to clinical heterogeneity. Meta-analysis was performed via Stata 17.0.

**Results:**

A total of 6680 patients were enrolled across 23 eligible trials. The results of the meta-analysis showed that teriparatide was superior to bisphosphonates in decreasing the risk of fracture (risk ratio (RR) = 0.61, 95% confidence interval (CI) (0.51, 0.74), *P* < 0.001). Denosumab showed no benefit compared to bisphosphonates in reducing the risk of fracture in treating osteoporosis (RR 0.99, 95% CI (0.62, 1.57), *P* = 0.96). Compared with bisphosphonates, teriparatide and denosumab could significantly improve femoral neck, total hip, and lumbar spine bone mineral density (BMD) (*P* < 0.05). Furthermore, teriparatide and denosumab did not increase the incidence of adverse events (teriparatide vs. bisphosphonates, RR 0.92, 95% CI (0.79, 1.08), *P* = 0.32; denosumab vs. bisphosphonates, RR 0.98, 95% CI (0.95, 1.02), *P* = 0.37).

**Conclusions:**

Teriparatide is superior to bisphosphonates in decreasing the risk of fracture in patients with osteoporosis. In addition, teriparatide and denosumab were more efficacious than bisphosphonates in increasing the percentage change in BMD at the femoral neck, total hip, and lumbar spine.

**Supplementary Information:**

The online version contains supplementary material available at 10.1007/s11657-024-01447-7.

## Introduction

Population aging is a widespread global phenomenon, leading to an increasing prevalence of diseases related to the elderly, with osteoporosis being a prominent example. Osteoporosis is a systemic bone disease characterized by decreased bone tissue microarchitecture and low bone mineral density, making bones more fragile and prone to fractures [1]. In the USA alone, approximately 1.5 million individuals experience fragility fractures annually [2], while osteoporosis affects nearly 90 million patients in China, with about 15% of individuals over the age of 50 suffering from fractures [3]. Fractures due to osteoporosis can significantly impact patients’ quality of life and lead to substantial financial burdens [4, 5]. The annual cost of fragility fractures in the United States is projected to rise by over 20%, reaching $25.3 billion by 2025 [6].The economic strain associated with osteoporosis underscores the urgent need for effective prevention and treatment strategies.

The primary strategies for osteoporosis prevention and treatment encompass fundamental measures, medicinal intervention, and rehabilitation. Clinical practice typically employs a range of pharmaceuticals, with bisphosphonates (BPs) having been the first-line treatment for osteoporosis since the 1990s [7]. Bisphosphonates work mainly by hampering osteoclast function to inhibit bone resorption [8]. After a 5-year treatment period with oral BPs or 3 years with intravenous BPs, a discontinuation phase may become necessary [9]. Currently, teriparatide is mostly used for patients with severe and high-risk osteoporosis [10]. Teriparatide increases bone formation by boosting the number of active osteoblasts while decreasing osteoblast apoptosis [11, 12]. Denosumab, a fully human monoclonal antibody, binds to the nuclear factor κB ligand-receptor activator (RANKL), which plays a vital role in osteoclast development, differentiation, and survival. This binding offers a possible treatment option for patients with osteoporosis [13]. However, due to the high side effects and poor adherence to anti-osteoporosis drugs, continuous research is needed to improve osteoporosis treatment strategies.

Numerous relevant meta-analyses that compared the effectiveness of teriparatide and denosumab with bisphosphonates have previously been published [14–19]. In studies by Yuan et al., the effectiveness of teriparatide vs. bisphosphonates in the treatment of postmenopausal osteoporosis was analyzed through a direct comparison [14]. Similarly, Lyu et al. employed a head-to-head comparison strategy to examine the clinical impacts of denosumab and bisphosphonates on osteoporosis patients [15, 16]. Furthermore, there have been network meta-analyses that indirectly assessed the effects of bisphosphonates, teriparatide, and denosumab [17–19].

However, these studies had some limitations. For instance, many of them exclusively focused on postmenopausal osteoporosis, thereby excluding male osteoporosis, and lacked a follow-up duration of at least 12 months. Most of the trial groups in these studies had already been treated with bisphosphonates, which hindered the direct distinction between the therapeutic effects of the different anti-osteoporosis medications. Moreover, these studies did not provide a comprehensive evaluation of teriparatide, denosumab, and bisphosphonates. For these reasons, we performed a systematic review and meta-analysis, including a direct comparison of randomized controlled trials (RCTs). Our methodology took into account the latest and high-quality RCTs and employed a wealth of data to validate our conclusions. In contrast to prior meta-analyses, this study was defined by its advanced evidence and the inclusion of a larger male patient population suffering from osteoporosis. We implemented new criteria, whereby participants in the experimental group refrained from bisphosphonate treatment and observed an extended follow-up period. In this study, we explored the influence of the three different anti-osteoporosis drugs (teriparatide, denosumab, and bisphosphonates) on fracture risk reduction, as well as the enhancement of femoral neck, total hip, and lumbar spine bone mineral density in patients with osteoporosis. Our goal was to provide the most comprehensive analysis available to patients.

## Materials and methods

The systematic review and meta-analysis were performed in line with the recommendations of the Cochrane Handbook and conducted in compliance with preferred Reporting Items for Systematic Reviews and Meta-Analyses (PRISMA) guidelines [20]. A formal protocol was enrolled on the International Prospective Register of Systematic Reviews (PROSPERO). Registration ID: CRD42023442508.

### Data sources and searches

Two authors systematically scanned published literature, we searched in PubMed, Embase, Cochrane Library, CNKI, SinoMed, VIP, and WanFang databases from inception to May 31, 2023. The retrieval strategy included a full-text search of Medical Subject Headings (MeSH) terms as well as word variations that have been searched. The following search terms were used: “osteoporosis” AND “Teriparatide” OR “Denosumab” [MeSH terms] AND Randomized controlled trial. Detailed term variations and complete search strategy results derived from the database can be seen in Table S1. References of all included articles were limited to English-language and Chinese-language studies. Besides, it only included randomized controlled trials, not review reports and animal trials. We also manually searched other electronic sources to identify other potentially eligible trials.

### Inclusion and exclusion criteria

Articles eligible for inclusion have satisfied the following criteria: (1) Population: Patients diagnosed with osteoporosis (*T*-score < − 2.5), no severe or long-term disabling conditions; (2) Intervention: Patients enrolled in the trial group received either teriparatide or denosumab; (3) Comparison: patients in the control group with bisphosphonates; (4) Outcomes: Percentage BMD change in femoral neck, total hip and lumbar spine, risk of fracture, and incidence of adverse events. Additionally, we included studies that were double-blind and open-label randomized controlled trials published in both English and Chinese languages. All included participants had a follow-up of at least 12 months and trials measuring at least one outcome of interest.

Exclusions from this study include (1) duplicate publication articles or studies from the same trials; (2) the type of articles was animal experiments, case report meta-analysis, and other non-RCTs; (3) the included population with cancer or glucocorticoid-induced patients; (4) osteoporotic patients with severe heart disease, severe liver disease, severe diabetes mellitus, severe renal impairment, etc. were excluded; (5) studied exclusion with participants who had previously combination or crossover treatment with bisphosphonates; (6) the follow-up time was less than 12 months; (7) articles exclusion with no outcome of interest and no original data from texts.

### Data retrieval and risk of bias assessment

Two reviewers independently screened and extracted the data using a predefined extraction form. In the first step, authors screened the title and abstracts from the retrieval literature and excluded articles if they failed to meet the eligibility criteria. Secondly, the authors browsed the full text, selecting the articles that met the qualified criteria and excluding the irrelevant ones. Then, performing data extraction and quantitative analysis from the included studies. If there is insufficient relevant data, reach out to the primary author of the article. In case the data is unavailable, utilize GetData software for extracting essential information from the figures. Any discrepancy or uncertain error was determined by means of discussion or a third reviewer check. Collected data contains the following parameters: the first name of the author, year of publication, country, basic treatment of included trials, gender, sample size, average age and body mass index of patients, intervention measures between the experimental group and control group, follow-up time, and outcomes. The first outcome was the incidence of risk of fracture; to confirm the influence of various factors on the fracture, we performed a subgroup analysis of this outcome according to the drug type of the control group, sample size, and follow-up time. Other outcomes included percent changes in bone mineral density in the femoral neck, total hip, lumbar spines, and adverse events.

Then, two reviewers independently assessed the risk of bias according to the Cochrane risk-of-bias tool [21]. If there was a dispute between the two authors, then disagreement was confirmed by discussion or consultation with a third author. The risk assessment includes the following items: (1) random sequence generation; (2) allocation concealment; (3) blinding of participants and personnel; (4) blinding of outcome assessment; (5) incomplete outcome data; (6) selective reporting; and (7) other bias. Each bias item was deemed to be high risk, unclear risk, or low risk in accordance with the bias score. The trials were deemed to possess a substantial risk of bias due to the assessment of one bias item as high risk, while all other items exhibited low risk of bias and some remained unclear. Besides, we drafted a funnel plot, Begg’s test, and Egger’s test to examine publication bias and further performed a heterogeneity test to assess the sensitivity of our meta-analysis.

### Data synthesis and statistical analysis

All data analyses were performed with Stata 17.0. Our determined outcomes were divided into two categories of data, one was continuous data (percent changes in femoral neck, total hip, lumbar spine BMD) and the other was dichotomous variables data (the risk of fracture and adverse events). Calculated using the weighted mean difference (WMD) with 95% confidence interval (CI) for continuous outcomes and the risk ratio (RR) with 95% CI for dichotomous outcomes. Possible heterogeneity between included studies, the *I*^2^ statistic is used to demonstrate the degree of heterogeneity: *I*^2^ > 50% reveals remarkable heterogeneity, outcomes data were pooled using a random-effect model; *I*^2^ ≤ 50% indicates slight heterogeneity, outcomes data were pooled using a fixed-effect model. To evaluate the deviation of included outcomes, publication bias was assessed by funnel plot, Begg’s test, and Egger’s test. All the tests were set at *P* < 0.05.

## Results

### Search results

**Fig. 1:**
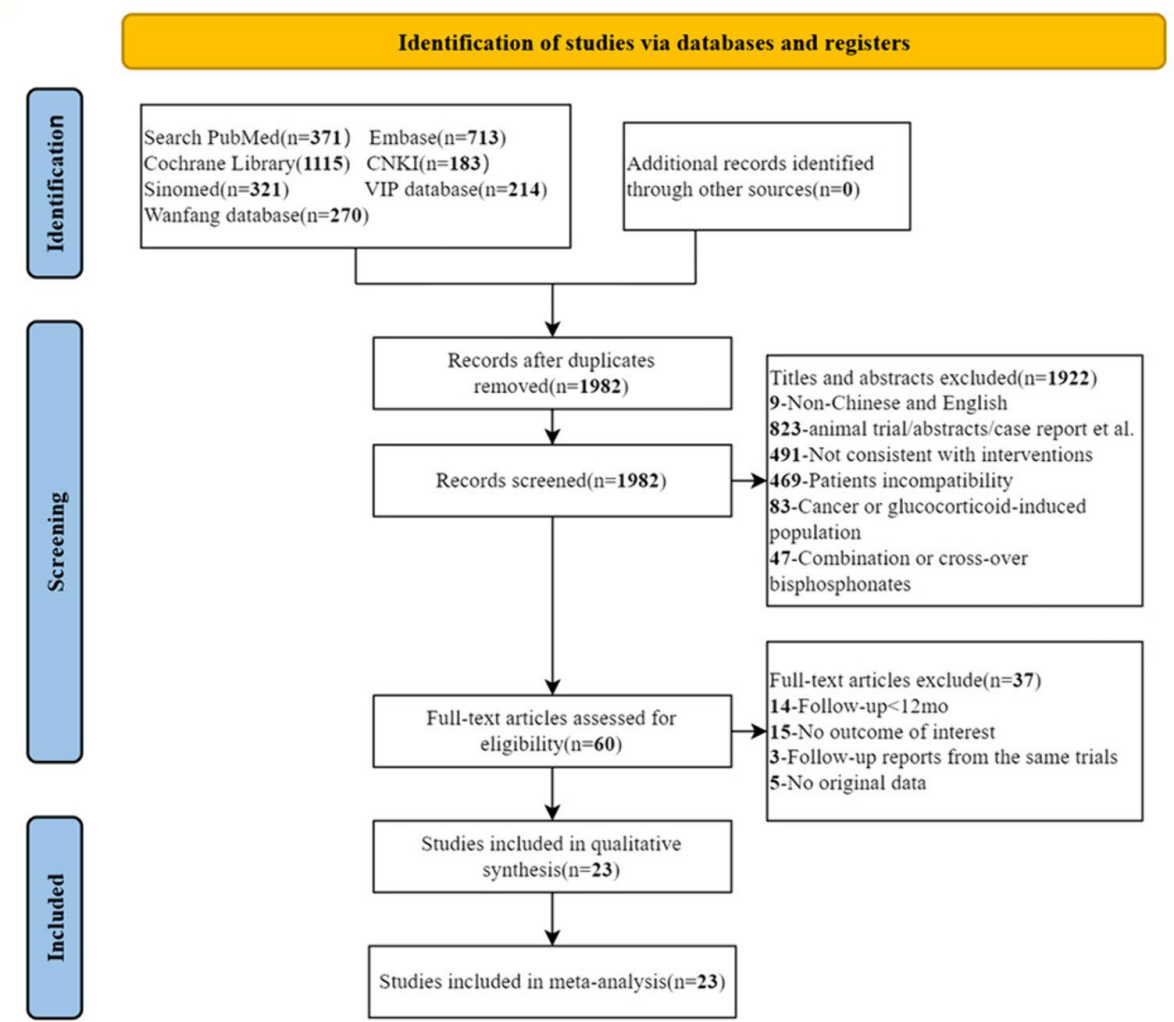
The flow chart of selection in this systematic review and meta-analysis

The process of literature screening met the PRISMA guideline flowchart (Fig. 1). An initial search for the effect of teriparatide or denosumab on osteoporosis identified 3187 articles: PubMed (371), Embase (713), Cochrane Library (1 115), CNKI (183), SinoMed (321), VIP database (214), and WanFang database (270). After the removal of duplicates and browsing the title and abstracts, 3127 articles were excluded, and 60 articles were possibly considered to meet the inclusion criteria. Finally, after screening the full texts, 37 studies were excluded based on the institutional inclusion//exclusion criteria, and 23 studies were selected for qualitative analysis and included in the meta-analysis.

### Trial characteristics of patients

The major baseline characteristics of the trials are provided in Tables 1 and 2. Included trials were published from 2002 to 2023 and contained a total of 6680 patients; there were 3679 patients in the experimental group and 3001 patients in the control group. All patients (mean age ranged from 51 to 77 years) were given supplemental treatment such as daily oral calcium and vitamin D. The sample size of subjects ranged from 9 to 680, follow-up time of trials ranged from 12 to 30 months. Among the included 23 trials, subjects of 17 trials were female osteoporosis, 2 trials were male osteopetrosis, and 4 trials were female and male osteoporosis. Of these, 16 of 23 trials compared teriparatide with bisphosphonates, and 7 of 23 trials compared denosumab with bisphosphonates. The bisphosphonates included risedronate, alendronate, and zoledronic acid.

**Table 1:** Basic characteristics of patients

Author	Year	Country	Sex (Female/Male)	Basic treatment	Follow-up (month)	Outcomes
Anastasilakis	2008	Greece	F	Calcium (500 mg/day), vitamin D (400 IU/day)	12	5
Body	2002	USA	F	Calcium (1000 mg/day), vitamin D (400–1200 IU/day)	12	12,345
Chiba	2022	Japan	F	Alfacalcidol (1 µg/day), vitamin D)	18	1235
Cosman	2011	USA	F	Calcium (1000–1200 mg/day), vitamin D (400–800 IU/day)	12	45
Finkelstein	2003	USA	M	Calcium (1000–1200 mg/day), vitamin D (400 IU/day)	30	123
Finkelstein	2010	USA	F	Calcium (1000–1200 mg/day), vitamin D (400 IU/day)	30	1235
Hadji	2012	Germany	F	Calcium (1000 mg/day), vitamin D (800 IU/day)	18	12,345
Keaveny	2007	USA	F	Calcium (1000 mg/day), vitamin D (400–800 IU/day)	18	13
Keaveny	2012	USA	F	Calcium (1000 mg/day), vitamin D (400–800 IU/day)	18	12
Kendler	2018	Canada	F	Calcium (500–1000 mg/day), vitamin D (400–800 IU/day)	24	45
Li	2022	China	F	Calcium (500 mg/day), vitamin D (200 IU/day)	12	345
Malouf-Sierra	2017	UK	F/M	Calcium (500–1000 mg/day), vitamin D (800 IU/day)	18	12,345
McClung	2005	Brazil	F	Calcium (1000 mg/day), vitamin D (400–800 IU/day)	18	45
Panico	2011	Italy	F	Calcium (1 g/day), vitamin D (800 IU/day)	18	1345
Walker	2013	Columbia	M	Calcium (500 mg/day), vitamin D (400 IU/day)	18	1345
Ji	2021	China	F	Calcium carbonate, calcitriol	12	2345
Beck	2008	USA	F	Calcium (1000 mg/day), vitamin D (400 IU/day)	24	1
Brown	2009	Spain	F	Calcium (500 mg/day), vitamin D (400 or 800 IU/day)	12	12,345
Iseri	2019	Japan	F/M	Calcitriol (0.25 µg/day), calcium lactate (1.5 g/day)	12	1345
Lewiecki	2007	USA	F	Calcium (1 g/day), vitamin D (400 IU/day)	24	45
McClung	2006	USA	F	Calcium (1 g/day), vitamin D (400 IU/day)	12	2345
Nakamura	2014	Japan	F/M	Calcium (600 mg/day), vitamin D (400 IU/day)	24	12,345
Nakura	2023	Japan	F/M	Calcium (600 mg/day), vitamin D (400 IU/day)	24	15

1, risk of fracture; 2, change in femoral neck BMD; 3, change in total hip BMD; 4, change in lumbar spine BMD; 5, adverse events

*SC* subcutaneous injection

**Table 2:** General characteristics of patients interventions

Author	Teriparatide OR denosumab							Bisphosphonates			
	No		Age (year)	BMI (kg/m^2^)			Intervention	No	Age (year)	BMI (kg/m^2^)	Intervention
Anastasilakis	22		65.4	28.2			SC teriparatide (20 µg/day)	22	64.7	28.1	Oral risedronate (35 mg/week)
Body	73		65	23.9			SC teriparatide (40 µg/day)	73	66	24.4	Oral alendronate (10 mg/day)
Chiba	46		71.7	23.4			SC teriparatide (20 µg/day)	40	71.9	23.1	Oral alendronate (35 mg/week)
Cosman	138		63.8	25.3			SC teriparatide (20 µg/day)	137	66.1	25.3	Intravenous zoledronic acid (5 mg/year)
Finkelstein	25		57	25.3			SC teriparatide (40 µg/day)	28	58	25.7	Oral alendronate (10 mg/day)
Finkelstein	20		65	24.9			SC teriparatide (40 µg/day)	29	64	25.6	Oral alendronate (10 mg/day)
Hadji	360		70.5	26.3			SC teriparatide (20 µg/day)	350	71.6	26.4	Oral risedronate (35 mg/week)
Keaveny	28		64.5	26.3			SC teriparatide (20 µg/day)	25	62.5	26.6	Oral alendronate (1 0 mg/day)
Keaveny	27		64.2	25.6			SC teriparatide (20 µg/day)	21	62.2	26.5	Oral alendronate (10 mg/day)
Kendler	680		72.6	26.9			SC teriparatide (20 µg/day)	680	71.6	21.7	Oral risedronate (35 mg/week)
Li	391		64.2	23.1			SC teriparatide (20 µg/day)	196	63.6	23.2	Oral alendronate (70 mg/week)
Malouf-Sierra	86		77.2	_			SC teriparatide (20 µg/day)	85	76.4	_	Oral risedronate (35 mg/week)
McClung		102	65.3		25.7	SC teriparatide (20 µg/day)		101	66.6	25.3	Oral alendronate (10 mg/day)
Panico		42	65		24.5	SC teriparatide (20 µg/day)		39	60	22.8	Oral alendronate (70 mg/week)
Walker		9	51.6		25.2	SC teriparatide (20 µg/day)		10	54	24.8	Oral risedronate (35 mg/week)
Ji		84	66.8		23.7	SC teriparatide (20 µg/day)		120	68.1	23.5	Intravenous zoledronic Acid (5 mg/year)
Beck		39	63		28.32	SC denosumab (60 mg/6 month)		38	63	27.39	Oral alendronate (70 mg/week)
Brown		594	64.1		_	SC denosumab (60 mg/6 month)		595	64.6	_	Oral alendronate (70 mg/week)
Iseri		24	71.3		19.9	SC denosumab (60 mg/6 month)		24	71.5	20.7	Intravenous alendronate (900 µg/4 week)
Lewiecki		319	62.3		_	SC denosumab (60 mg/6 month)		47	62.8	_	Oral alendronate (70 mg/week)
McClung		47	63.1		15.7	SC denosumab (60 mg/6 month)		47	62.8	13.7	Oral alendronate (70 mg/week)
Nakamura		472	69.9		22.6	SC denosumab (60 mg/6 month)		242	70.2	22.3	Oral risedronate (35 mg/week)
Nakura		51	69.9		23.2	SC denosumab (60 mg/6 month)		52	68.3	22.5	Oral risedronate (17.5 mg/week)

### Risk of bias assessment

**Fig. 2:**
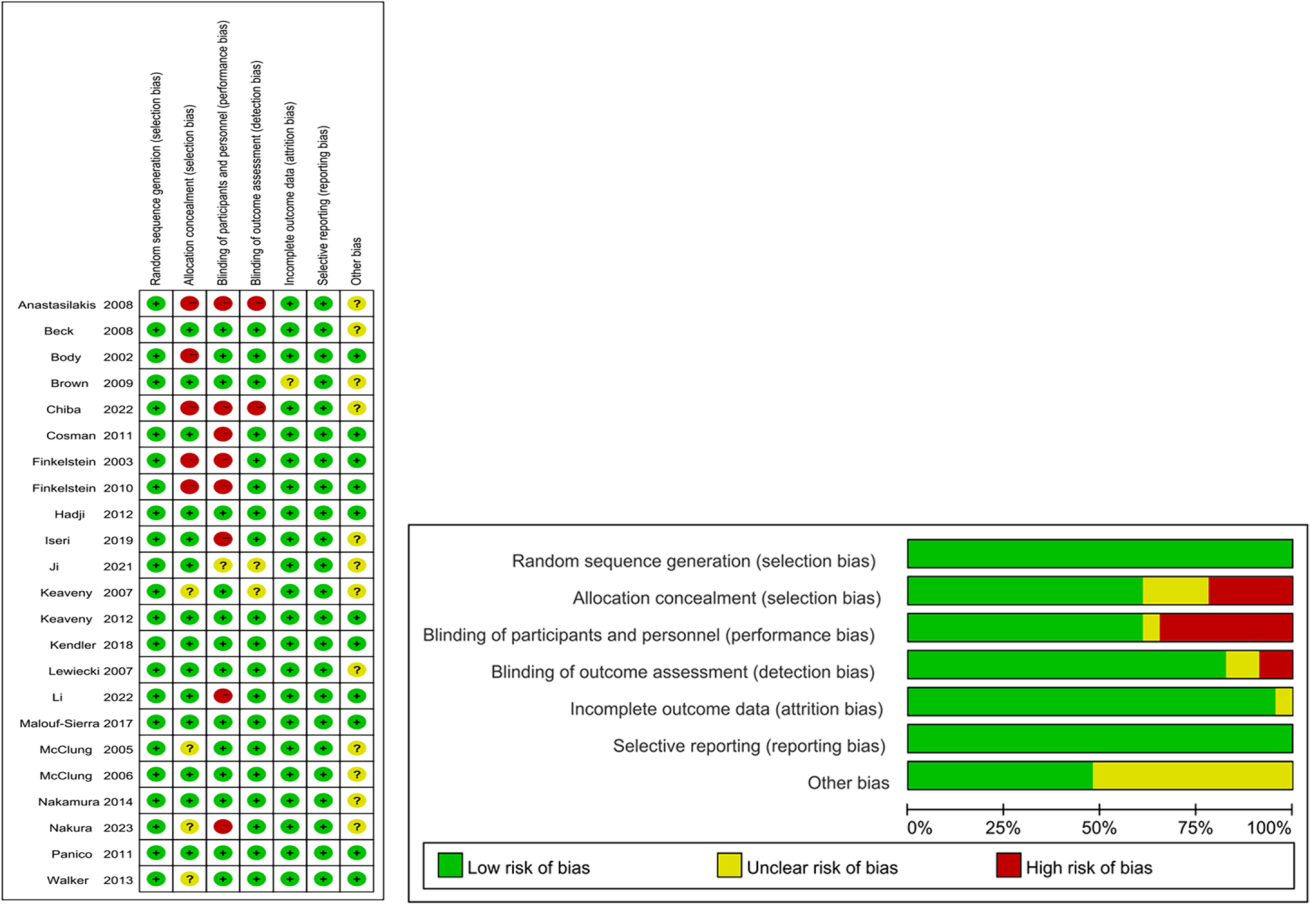
Assessment of risk of bias. Green, low risk of bias; yellow, unclear risk of bias; red, high risk of bias

Assessment of the risk of bias was summarized in Fig. 2. Overall, five trials were well-arranged with a low risk of bias, nine trials in unclear, and nine trials in high risk of bias. Random sequence generation and selective reporting were reported and sorted as being at low risk of bias in all trials. Blinding of outcome assessment and incomplete outcome data were generated in over 75% of these trials. Fourteen trials reported appropriate allocation concealment and the assessment of blinding of participants was unclear or inadequately reported in eight trials.

### Results of meta-analysis

#### Risk of fractures

The analysis included 15 trials involving 6264 patients that assessed the risk of fracture incidence. This encompassed 10 trials with 3602 patients comparing teriparatide directly with bisphosphonates, as well as 5 trials with 2662 patients comparing denosumab with bisphosphonates [22–36].

**Fig. 3:**
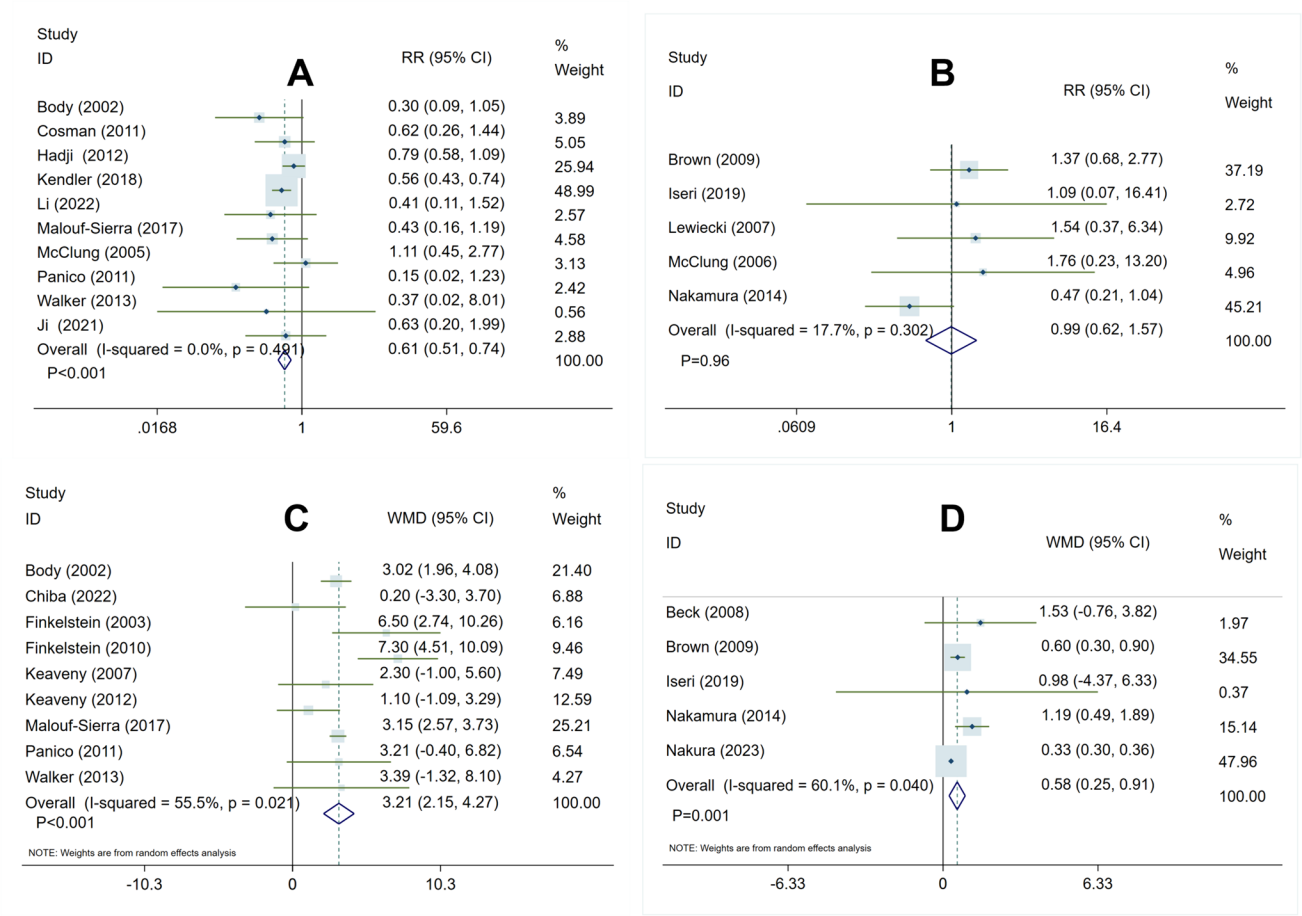
Forest plot of meta-analysis result in risk of fractures and percent changes at femoral neck BMD. **A** Teriparatide vs. bisphosphonates in risk of fractures. **B** denosumab vs. bisphosphonates in risk of fractures. **C** Teriparatide vs. bisphosphonates in percent changes at femoral neck BMD. **D** Denosumab vs. bisphosphonates in percent changes at femoral neck BMD

In the comparison of teriparatide vs. bisphosphonate treatment, the heterogeneity test yielded the following results:* I*^2^ = 0.0%, *P* = 0.49, indicating a lack of significant heterogeneity among the studies. This suggested the use of a fixed-effect model for analysis. The pooled analysis demonstrated that teriparatide treatment, in comparison to bisphosphonates, significantly reduced the risk of fracture occurrence (RR 0.61, 95% CI 0.51–0.74, *P* < 0.001) (Fig. 3A).

In the comparison of denosumab vs. bisphosphonate treatment, the heterogeneity test yielded the following results:* I*^2^ = 17.7%, *P* = 0.30, indicating no significant heterogeneity among the trials. This suggested the utilization of a fixed-effect model for analysis. The pooled analysis indicated that denosumab had a similar impact to bisphosphonates in reducing the occurrence of fracture risk (RR 0.99, 95% CI 0.62–1.57, *P* = 0.96) (Fig. 3B).

#### Percent changes at femoral neck BMD

In this analysis, percent changes at femoral neck BMD were assessed in 14 trials comprising 2706 patients with 9 trials involving 611 patients comparing teriparatide and bisphosphonates, and 5 trials involving 2095 patients comparing denosumab and bisphosphonates [22, 28, 30–33, 36–43].

When comparing teriparatide treatment with bisphosphonates, the heterogeneity test yielded the following results: *I*^2^ = 55.5%, *P* < 0.05, indicating a slight degree of heterogeneity among the trials. Subsequently, a sensitivity analysis was conducted by systematically removing one study at a time, which confirmed the stability of the included studies (Fig. S1) and recommended the use of a random-effect model for the analysis. The pooled analysis revealed that teriparatide treatment, in comparison to bisphosphonates, resulted in a further increase in percent changes in femoral neck BMD (WMD 3.21, 95% CI 2.15–4.27, *P* = 0.001) (Fig. 3C).

After conducting a heterogeneity test, it was found that there was slight heterogeneity among the included studies comparing denosumab with bisphosphonates, with *I*^2^ = 60.1% and *P* < 0.05. To ensure data accuracy, a sensitivity analysis was performed by systematically excluding one study at a time. The results indicated that the included studies demonstrated stability (Fig. S2), leading to the recommendation of using a random-effect model for the analysis. The pooled analysis revealed that denosumab was more effective than bisphosphonates in increasing the percent changes at femoral neck BMD (WMD 0.58, 95%CI 0.25–0.91, *P* = 0.001) (Fig. 3D).

#### Percent changes at total hip BMD

In this analysis, 11 trials involving 3175 subjects reported percent changes in total hip BMD. The teriparatide vs. bisphosphonates group comprised 8 RCTs with 1190 subjects, while the denosumab vs. bisphosphonates group included 3 RCTs with 1985 subjects [22, 24, 25, 32, 36–41, 43].

**Fig. 4:**
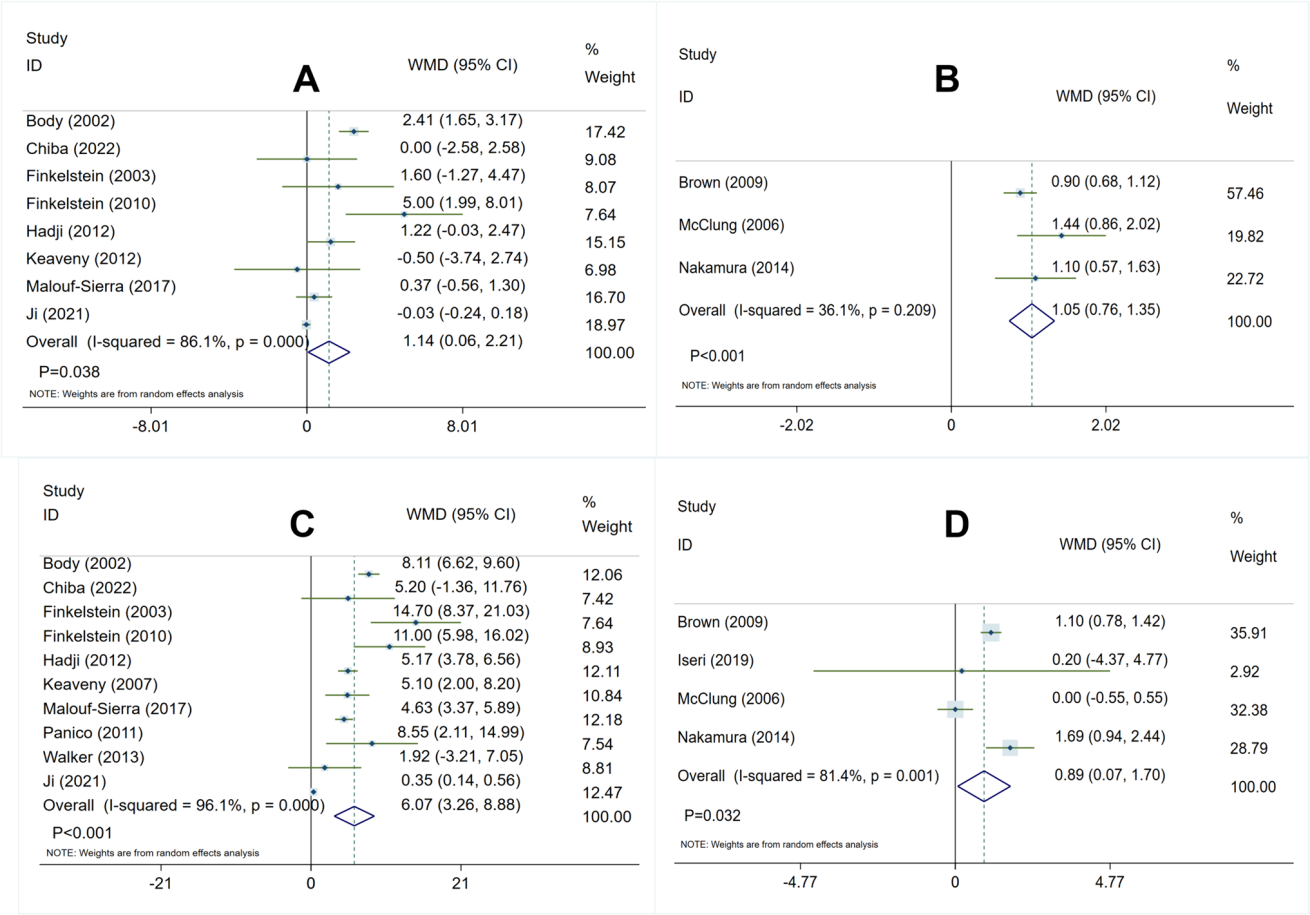
Forest plot of meta-analysis result in percent changes at total hip and lumbar spine BMD. **A** Teriparatide vs. bisphosphonates in percent changes at total hip. **B** Denosumab vs. bisphosphonates in percent changes at total hip. **C** Teriparatide vs. bisphosphonates in percent changes at lumbar spine BMD. **D** Denosumab vs. bisphosphonates in percent changes at lumbar spine BMD

In the comparison between the teriparatide group and the bisphosphonates group, the heterogeneity test yielded as follows:* I*^2^ = 86.1%, *P* < 0.05, indicating potential high heterogeneity among the included trials. To ensure the accuracy of the study, a sensitivity analysis was conducted where each study was systematically excluded to enhance the stability of the results (Fig. S3). A random-effect model was utilized for analysis. The overall pooled analysis revealed that teriparatide was significantly more effective than bisphosphonates in increasing percent changes in total hip BMD (WMD 1.14, 95% CI 0.06–2.21, *P* = 0.038) (Fig. 4A).

In the comparison between the denosumab group and the bisphosphonates group, the heterogeneity test revealed as follows: *I*^2^ = 36.1%, *P* = 0.21, indicating no significant heterogeneity among the trials. A fixed-effect model was employed for analysis. The overall pooled analysis showed that denosumab was significantly more effective than bisphosphonates in increasing percent changes in total hip BMD (WMD 1.05, 95% CI 0.76–1.35, *P* < 0.001) (Fig. 4B).

#### Percent changes at lumbar spine BMD

A total of 14 trials with 3332 subjects, comprising 10 randomized controlled trials involving 1301 subjects comparing teriparatide and bisphosphonates, and 4 RCTs involving 2031 subjects comparing denosumab and bisphosphonates, reported percent changes in total hip BMD [22, 24, 25, 28, 30–33, 35–40].

In the comparison between teriparatide and bisphosphonates treatment, the heterogeneity test showed as follows: *I*^2^ = 96.1%, *P* < 0.05, indicating potential heterogeneity among the randomized controlled trials. Therefore, a sensitivity analysis was performed by leaving out one study in turn, and results showed these RCTs have better stability (Fig. S4). Then, a random-effect model was utilized for analysis. The overall pooled analysis demonstrated that compared with bisphosphonates, teriparatide significantly improved percent changes at lumbar spine BMD (WMD 6.07, 95% CI 3.26–8.88, *P* < 0.001) (Fig. 4C).

In the comparison between denosumab and bisphosphonates treatment, the heterogeneity test showed as follows: *I*^2^ = 81.4%, *P* < 0.05, suggesting potential heterogeneity among the RCTs. A sensitivity analysis was conducted to verify the accuracy of the included studies by sequentially omitting one study at a time, resulting in better stability in the included trials (Fig. S5), therefore implying the use of a random-effects model for analysis. The overall pooled analysis demonstrated that denosumab treatment significantly increased percent changes in lumbar spine bone mineral density compared to bisphosphonates (WMD 0.89, 95% CI 0.07–1.70, *P* = 0.032) (Fig. 4D).

### Incidence of adverse events

The analysis included a total of 19 trials with 6221 patients assessing the incidence of adverse events, comprising 13 RCTs with 4010 patients comparing teriparatide with bisphosphonates, and 6 RCTs with 2211 patients comparing denosumab with bisphosphonates [22–37, 39, 43, 44]. The adverse included nausea, pyrexia, back pain, arthralgia, myalgia, skin injury, and leg cramps.

**Fig. 5:**
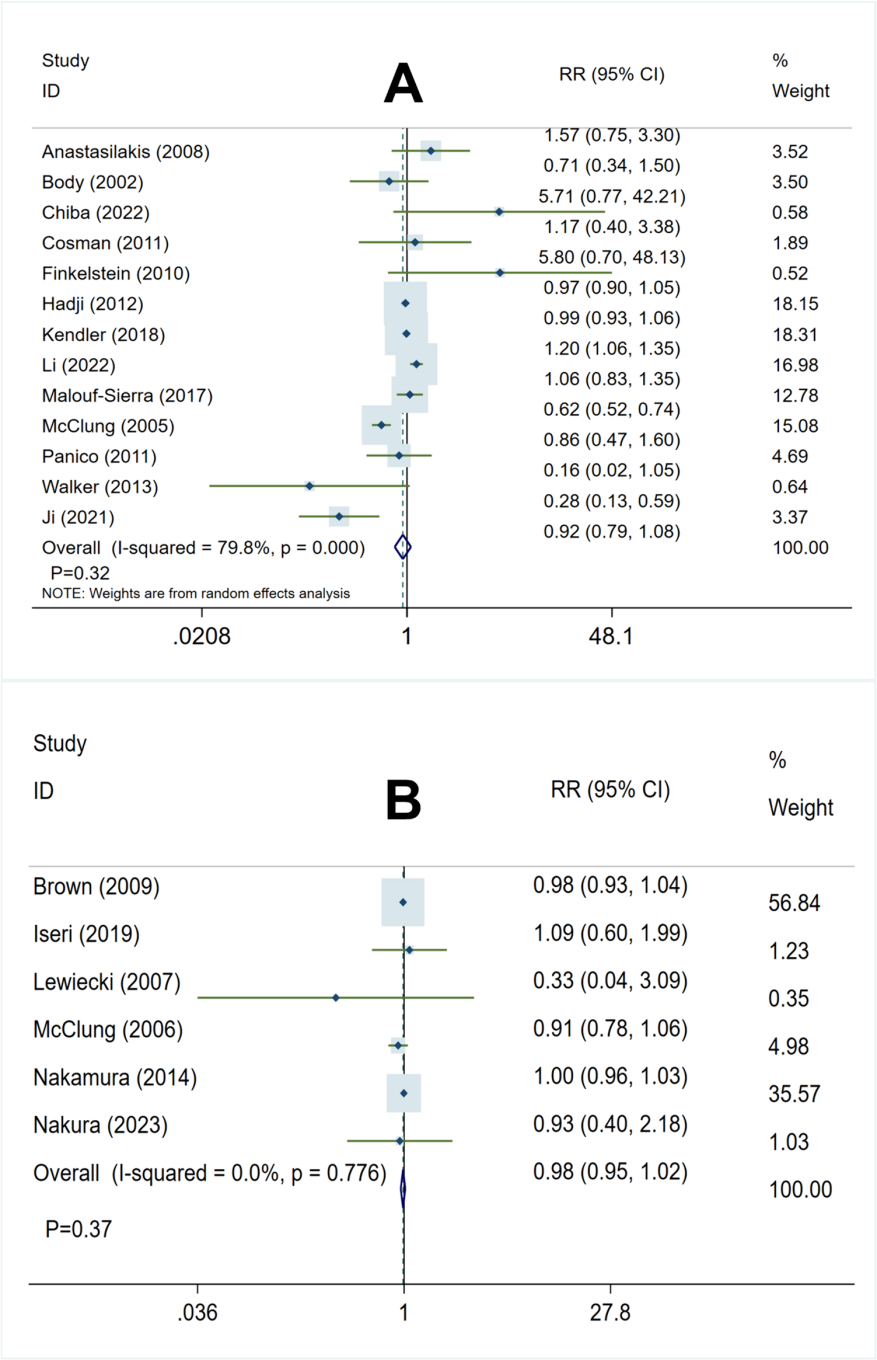
Forest plot of meta-analysis result in incidence of adverse. **A** Teriparatide vs. bisphosphonates in incidence of adverse. **B** Denosumab vs. bisphosphonates in incidence of adverse

In comparison between the teriparatide group and bisphosphonates group, the heterogeneity test showed as follows: *I*^2^ = 79.8%, *P* < 0.05, suggesting potential heterogeneity among studies, then we performed a sensitivity analysis to ensure study accuracy, leaving out one study in turn, and results found included studies had better stability (Fig. S6), therefore implying the use of a random-effects model for analysis. The overall pooled analysis has shown that teriparatide and bisphosphonates were similar in the incidence of adverse events (RR 0.92, 95% CI 0.79–1.08, *P* = 0.32) (Fig. 5A).

Comparison of the denosumab and bisphosphonates groups revealed the following results from the heterogeneity test:* I*^2^ = 0.0%, *P* = 0.78. These findings indicate that no significant heterogeneity was present among the studies. Therefore, a fixed-effect model was utilized for the analysis. The overall pooled analysis has shown that denosumab and bisphosphonates were equal in the incidence of adverse events (RR 0.98, 95% CI 0.95–1.02, *P* = 0.37) (Fig. 5B).

### Subgroup analysis

Performed subgroup analysis on decreasing the incidence of risk of fracture according to types of bisphosphonates in the control group, sample size, and follow-up time (Tables 3 and 4). For comparison of teriparatide and bisphosphonates, when the control group was treated with alendronate and risedronate, the experimental group could significantly decrease the incidence of risk of fracture. However, compared with zoledronic acid, teriparatide has no obvious advantage (RR 0.62, 95% CI 0.31–1.23, *P* = 0.17). Subgroup analysis of sample size and follow-up time was consistent with previous results. For comparison of denosumab and bisphosphonates, the findings of all subgroup analyses that reported denosumab and bisphosphonates were not significantly different regarding the incidence of risk of fracture.

**Table 3:** Subgroup analysis for teriparatide vs. bisphosphonates for risk of fracture

Subgroup	No. trials	*P* value	RR (95% CI)	*I*-squared
Drug of bisphosphonates				
Alendronate	4	0.022	0.51 (0.28, 0.91)	38.00%
Risedronate	4	0.000	0.63 (0.51, 0.77)	10.30%
Zoledronic acid	2	0.173	0.62 (0.31, 1.23)	0.00%
Sample size of participants				
No. (≤ 100 of participants)	4	0.008	0.37 (0.18, 0.77)	0.00%
No. (> 100 of participants)	6	0.000	0.64 (0.53, 0.77)	1.30%
Follow-up				
= 12 months	4	0.011	0.50 (0.29, 0.85)	0.00%
= 18 months	5	0.027	0.73 (0.55, 0.96)	10.90%
= 24 months	1	0.000	0.56 (0.43, 0.74)	_

**Table 4:** Subgroup analysis for denosumab vs. bisphosphonates for risk of fracture

Subgroup	No. trials	*P* value	RR (95% CI)	*I*-squared
Drug of bisphosphonates				
Alendronate	4	0.242	1.42 (0.79, 2.56)	0.00%
Risedronate	1	0.063	0.47 (0.21, 1.04)	_
Sample size of participants				
No. (≤ 100 of participants)	3	0.434	1.53 (0.53, 4.45)	0.00%
No. (> 100 of participants)	2	0.605	0.87 (0.52, 1.46)	74.30%
Follow-up				
= 12 months	3	0.313	1.39 (0.73, 2.66)	0.00%
= 24 months	2	0.229	0.66 (0.34, 1.30)	52.00%

### Publication bias

**Fig. 6:**
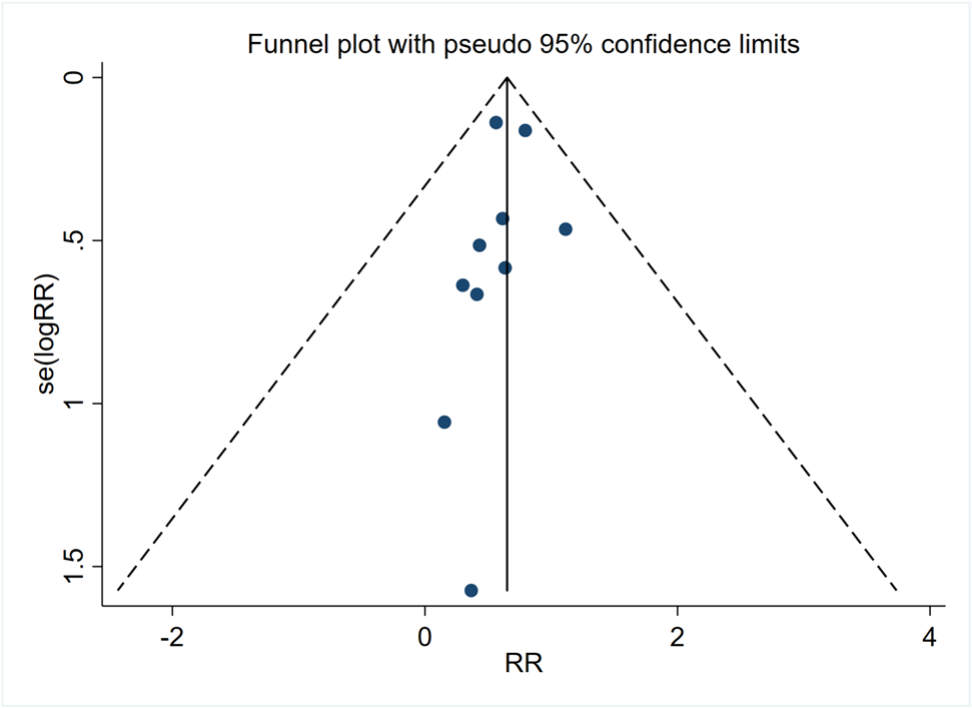
Funnel plot of risk of fracture

To access the publication bias of the 10 trials in this meta-analysis, compared teriparatide and bisphosphonates on the risk of fracture, there was no significant publication bias according to the examination of the funnel plot (Fig. 6), and further, the Begg’s test yielded *P* = 0.28 (Fig. S7) and Egger’s test yielded *P* = 0.24 (Fig. S8).

## Discussion

In clinical practice, selecting the appropriate anti-osteoporosis drug treatment is vital for patients, given that long-term medication is necessary to delay disease progression and improve the quality of life. Effective anti-osteoporosis drug therapy increases bone density, improves bone quality, and significantly reduces the risk of fractures. However, due to limitations in the effectiveness of the original medication used, patients often need to switch to other medications. For example, romosozumab, zoledronic acid, teriparatide, denosumab, and elcatonin are often used clinically; however, patients often need to be switched to other medications due to the limited efficacy of the original medications used. Therefore, this work focuses on comparing the efficacy and safety of different anti-osteoporosis drugs in osteoporosis not previously treated with bisphosphonates.

After carefully examining 23 randomized controlled trials involving 6680 patients, our meta-analysis compared the effectiveness and safety of teriparatide and bisphosphonates, denosumab, and bisphosphonates in treating osteoporosis. The results indicated that teriparatide significantly reduced the risk of fractures compared to bisphosphonates. Furthermore, both teriparatide and denosumab surpass bisphosphonates in improving bone mineral density in the femoral neck, total hip, and lumbar spine. There are no notable differences found in the incidence of adverse events between teriparatide, denosumab, and bisphosphonates.

Though several relevant meta-analyses have been previously published, there were several points of differences between our meta-analysis and those of earlier studies [14–19]. Firstly, two prior meta-analyses were conducted solely on postmenopausal women [14, 16]. While the prevalence of osteoporosis is significantly higher in women, the likelihood of osteoporotic fractures in men is comparable to that in women. Hence, attention must also be directed at preventing and treating osteoporosis in men. Our meta-analysis includes both men and women suffering from osteoporosis, thus broadening the scope of the population studied and increasing the reliability of the results. Secondly, in a meta-analysis comparing denosumab and bisphosphonates, patients in the denosumab group had previously received bisphosphonate treatment [15]. However, the patients in our denosumab group had not received bisphosphonate treatment, leading to a significant reduction in the heterogeneity of the meta-analysis. Thirdly, several orthodox meta-analyses have compared the effectiveness and safety of anti-osteoporosis drugs in treating osteoporosis [17–19]. However, the comparison among teriparatide, denosumab, and bisphosphonates in those studies was indirect head-to-head comparisons. Moreover, in our study, the osteoporosis patients were under treatment for at least 12 months. We also excluded patients with osteoporosis induced by malignant diseases or those taking other hormone drugs, allowing us to effectively evaluate the drug treatment for patients suffering from age-induced osteoporosis. In summary, our meta-analysis is the most comprehensive comparison of the efficacy of teriparatide, denosumab, and bisphosphonates, including the latest and high-quality randomized controlled trials.

In this systematic review and meta-analysis, a total of 15 randomized controlled trials showed that teriparatide and denosumab are more effective than bisphosphonates in increasing the bone mineral density (BMD) in the femoral neck. Eleven RCTs demonstrated that teriparatide and denosumab are more effective than bisphosphonates in improving the BMD of the total hip, and 14 RCTs reported that teriparatide and denosumab are beneficial in enhancing lumbar spine BMD. This suggests that teriparatide and denosumab can be selected to augment the body’s bone mineral density and restore it to normal levels in patients with low bone mass. Fifteen RCTs included in this meta-analysis compared the three kinds of anti-osteoporosis drugs in reducing the risk of fractures, revealing that teriparatide outperformed bisphosphonates after 12, 18, and 24 months of treatment. This suggests that teriparatide could be preferentially considered in the clinical treatment of patients with extremely high fracture risk. Compared with bisphosphonates, denosumab did not yield any significant effect on fractures for the following reasons. Firstly, the lack of sequential or combination treatment with bisphosphonate may explain why the benefit of denosumab is not apparent. Consequently, it can be inferred that denosumab needs to be used for a long period before it can play a pivotal role in reducing the risk of fractures. In addition to this, it has also been shown in several studies that sequential treatment with bisphosphonates followed by denosumab helps to reduce fractures, and therefore, based on the findings of this study, it is suggested that sequential treatment with denosumab and bisphosphonates in patients with osteoporosis is more effective in achieving a reduction in fractures [45–47]. Secondly, the number of patients included in the RCTs of the denosumab group and the bisphosphonate group was small, leading to unclear effects. Hence, more research is needed to validate this hypothesis. The results from subgroup analyses showed that teriparatide surpassed alendronate and risedronate in reducing the risk of fractures, but there was no significant difference between teriparatide and zoledronic acid. This suggested that zoledronic acid may be very effective in reducing the risk of fractures among bisphosphonates. Therefore, using teriparatide and denosumab in clinical practice greatly benefits the treatment of patients suffering from osteoporosis. The safety of drugs for treating osteoporosis was also examined, with results indicating that all three types of drugs did not have significant differences in terms of side effects. Therefore, when it comes to selecting an anti-osteoporosis medication for treating osteoporosis, the priority should be to choose drugs that demonstrate significant effectiveness and minimal adverse effects.

Despite these findings, several limitations must be taken into account. Firstly, two different doses of teriparatide were used in this systematic review and meta-analysis—20 µg and 40 µg. Most of the studies used 20 µg of teriparatide. As such, no comparisons were conducted regarding the efficacy of varying doses of teriparatide and bisphosphonates in reducing fracture risks, enhancing bone mineral density, and decreasing the occurrence of adverse events. Secondly, the heterogeneity of the study could be ascribed to the type of bisphosphonates used, different follow-up durations, and adjuvant therapy. And there is an impact of RCT studies with small samples on the results, and more and larger sample sizes are needed for validation. Thirdly, the evaluation of drug efficacy in this meta-analysis only considered changes in bone mineral density and risk of fracture as observational indicators, ignoring other indicators such as bone metabolism markers. Fourthly, this study did not compare the serious adverse reactions caused by teriparatide, denosumab, and bisphosphonates. Finally, in subgroup analyses, there was no significant difference in fracture reduction between zoledronic acid and teriparatide. However, as only two RCTs were included for comparison, further studies are required to confirm whether teriparatide indeed surpasses zoledronic acid in terms of its fracture reduction efficacy.

## Conclusions

The results of our research reveal that teriparatide and denosumab outperform bisphosphonates in increasing percentage changes in femoral neck, total hip, and lumbar spine BMD for patients suffering from osteoporosis. Additionally, teriparatide significantly reduces the risk of fractures compared to bisphosphonates. Existing data suggests no substantial difference in the incidence of adverse events among teriparatide, denosumab, and bisphosphonates. As such, both teriparatide and denosumab have proven to be effective and do not result in additional adverse events when treating osteoporosis patients. However, additional randomized controlled trials are still required for further confirmation.

## Supplementary Information

Below is the link to the electronic supplementary material.

Supplementary file1 (PDF 757 KB)

## Data Availability

The datasets for this study can be found in the article/Supplementary Material. Further inquiries can be directed to the corresponding author.
