# Correction: Efficacy and safety of teriparatide vs. bisphosphonates and denosumab vs. bisphosphonates in osteoporosis not previously treated with bisphosphonates: a systematic review and meta-analysis of randomized controlled trials

**DOI:** 10.1007/s11657-024-01478-0

**Published:** 2024-11-21

**Authors:** Mingnian Li, Zhuoqi Ge, Benqi Zhang, Li Sun, Zhongyuan Wang, Tao Zou, Qi Chen

**Affiliations:** 1 College of Pharmacy, Guizhou Medical University, Guiyang, Guizhou China; 2 Department of Pharmacy, Guizhou Provincial People’s Hospital, Guiyang, Guizhou China; 3 Department of Pharmacy, Beijing Jishuitan Hospital Guizhou Hospital, Guiyang, Guizhou China

**Correction: Archives of Osteoporosis (2024) 19:89**

10.1007/s11657-024-01447-7

In this article the affiliation details for Author Mingnian Li were incorrectly given as 'Department of Pharmacy, Guizhou Provincial People’s Hospital, Guiyang, Guizhou, China' but should have been 'College of Pharmacy, Guizhou Medical University, Guiyang, Guizhou, China'.

The corrected affiliation details are as follows:

Mingnian Li^1^ · Zhuoqi Ge^2^ · Benqi Zhang^1^ · Li Sun^2^ · Zhongyuan Wang^2^ · Tao Zou^3^ · Qi Chen^1,2^

^1^ College of Pharmacy, Guizhou Medical University, Guiyang, Guizhou, China.

^2^ Department of Pharmacy, Guizhou Provincial People’s Hospital, Guiyang, Guizhou, China.

^3^ Department of Pharmacy, Beijing Jishuitan Hospital Guizhou Hospital, Guiyang, Guizhou, China.

The original article has been corrected.

