# Supplementary material for: Efficacy and safety of teriparatide vs. bisphosphonates and denosumab vs. bisphosphonates in osteoporosis not previously treated with bisphosphonates: a systematic review and meta-analysis of randomized controlled trials
Source: Arch Osteoporos. 2024 Sep 23;19(1):89. doi: 10.1007/s11657-024-01447-7 (PMC11420281; doi:10.1007/s11657-024-01447-7)
Supplement: Supplementary file 1 — Supplementary file1 (PDF 757 KB) [file 11657_2024_1447_MOESM1_ESM.pdf]

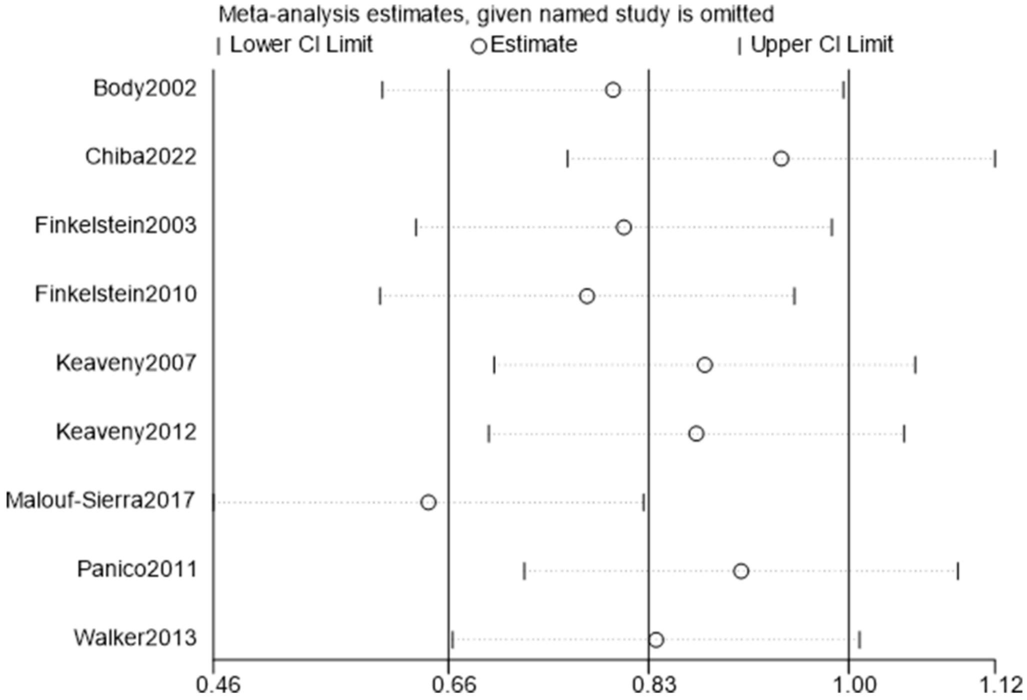

Fig. S1. Sensitivity analysis for comparing teriparatide versus bisphosphonates in percent changes at femoral neck BMD.

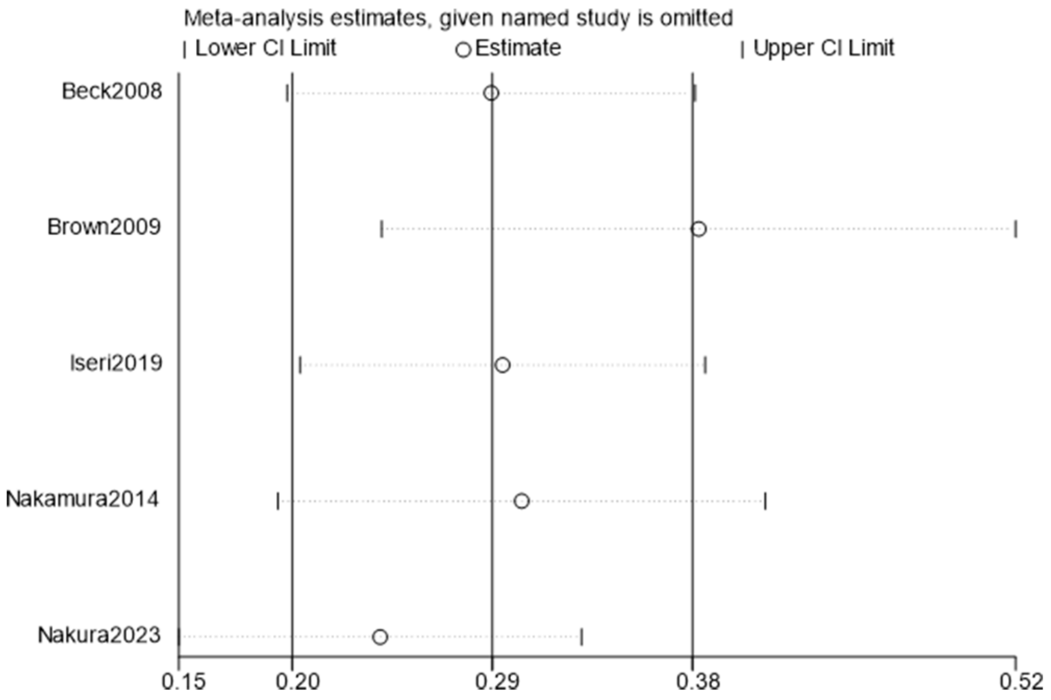

Fig. S2. Sensitivity analysis for comparing denosumab versus bisphosphonates in percent changes at femoral neck BMD.

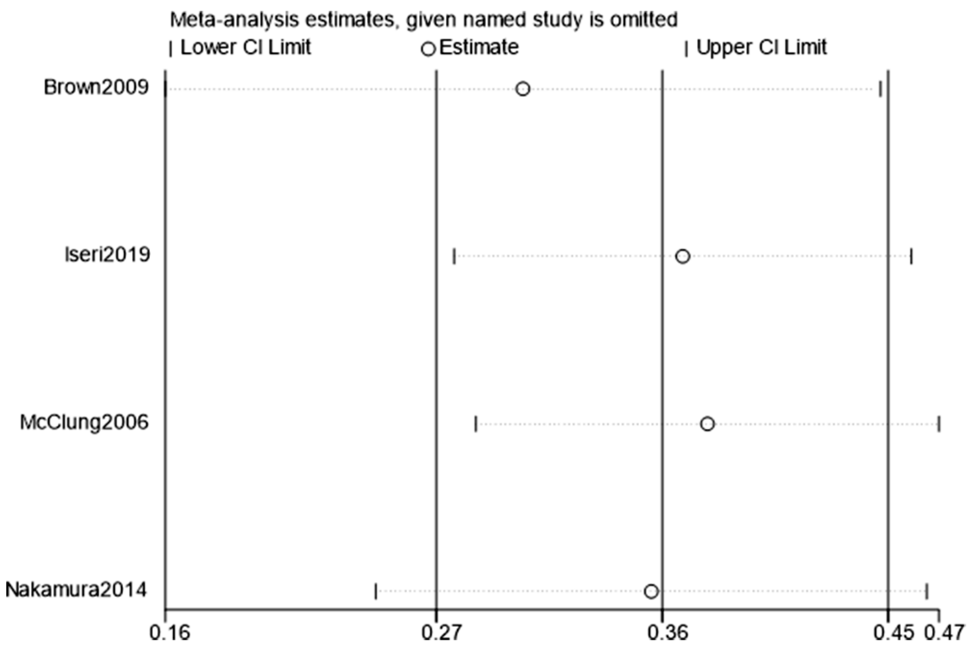

Fig. S3. Sensitivity analysis for comparing teriparatide versus bisphosphonates in percent changes at total hip BMD.

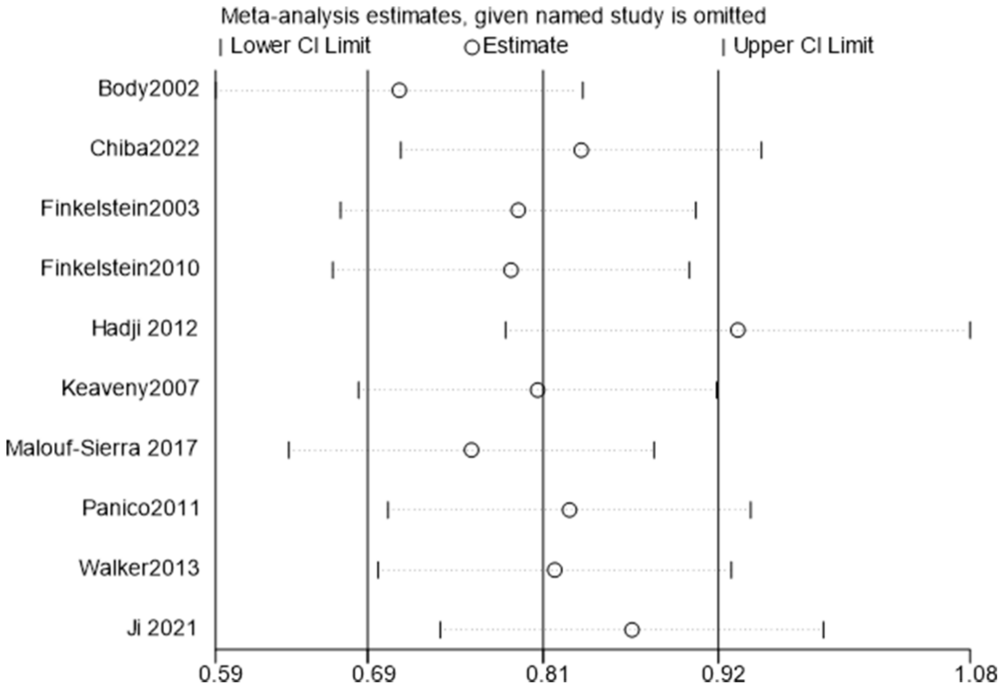

Fig. S4. Sensitivity analysis for comparing teriparatide versus bisphosphonates in percent changes at lumbar spine BMD.

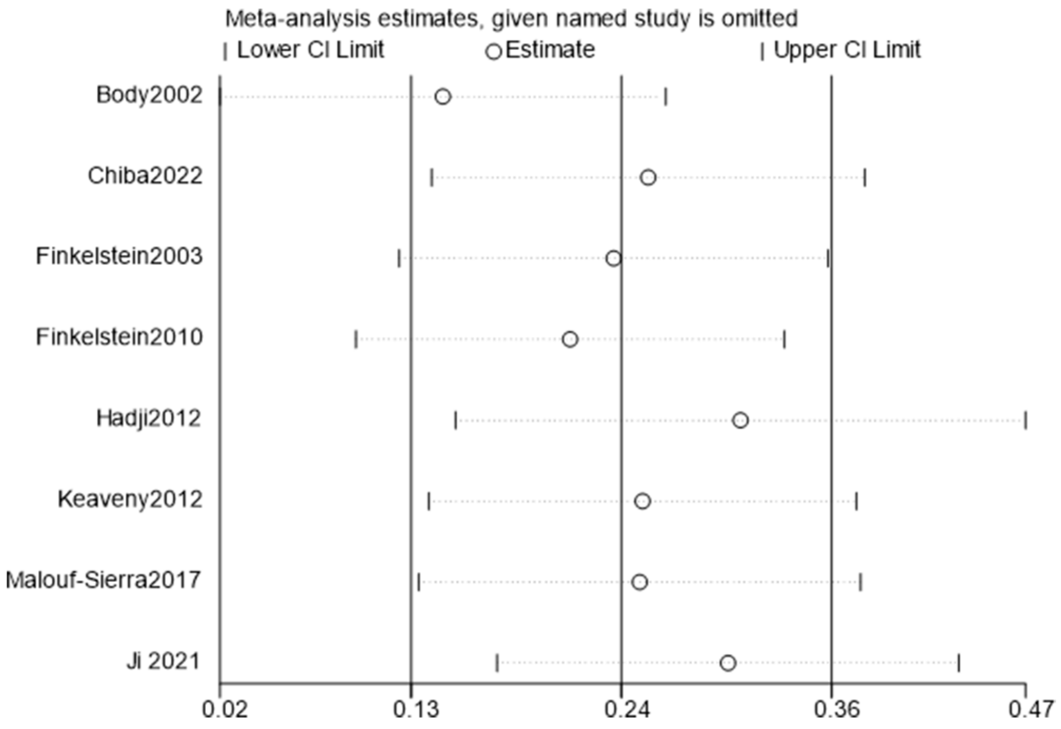

Fig. S5. Sensitivity analysis for comparing denosumab ver-sus bisphosphonates in percent changes at lumbar spine BMD.

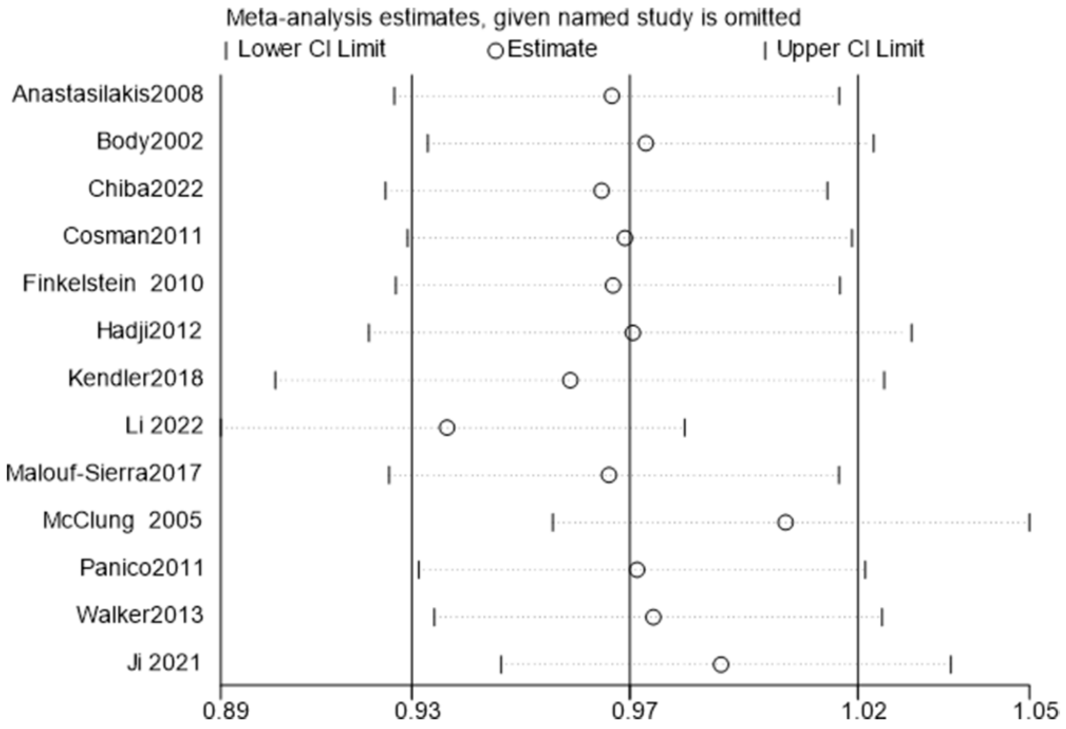

Fig. S6. Sensitivity analysis for comparing teriparatide versus bisphosphonates in the incidence of adverse events.

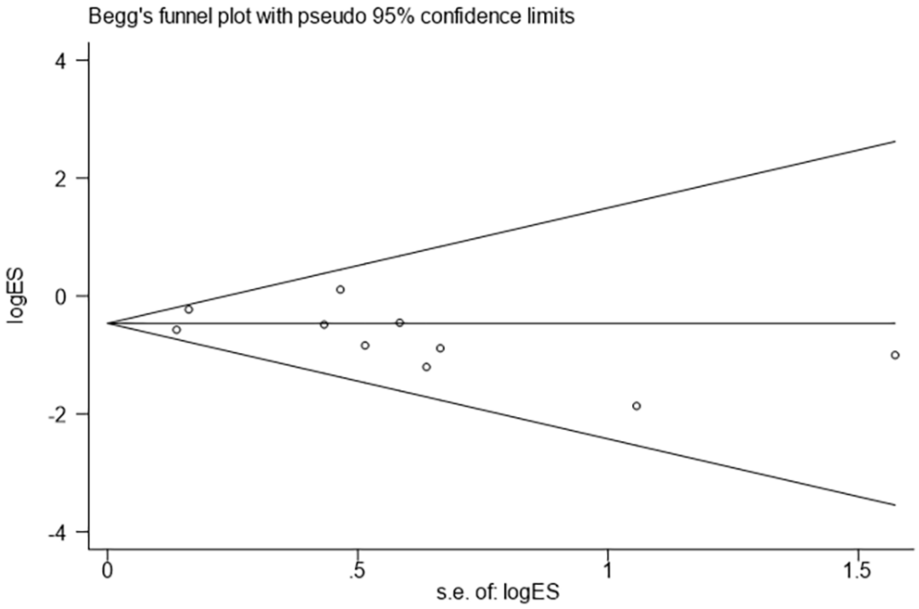

Fig. S7. Begg’s test for incidence of risk of fracture. Fig. S8. Egger’s test for incidence of risk of fracture.

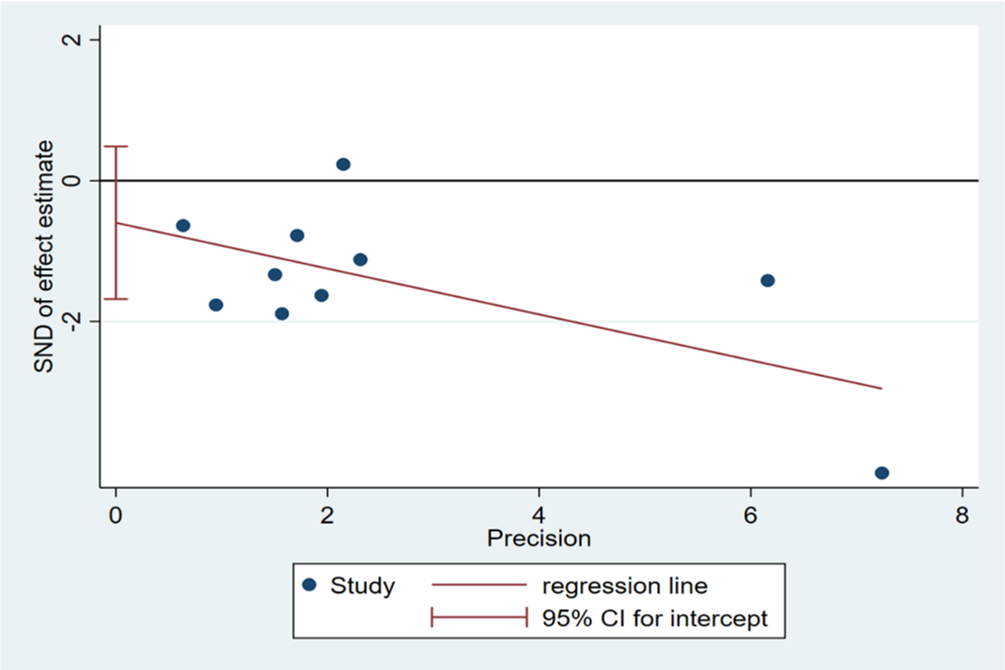

Fig. S8. Egger's test for incidence of risk of fracture.

TABLES 1. Search Strategy

Database	Search Strategy	Results
PubMed	#1 “Osteoporosis” [Mesh]<br>#2 “Osteoporosis entry terms” [All fields]<br>#3 #1 OR#2<br>#4 “Teriparatide” [Mesh]<br>#5 “Teriparatide entry terms” [All fields]<br>#6 #4 OR#5<br>#7“Denosumab” [Mesh]<br>#8 “Denosumab entry terms” [All fields]<br>#9 #7 OR#8<br>#10 #6 OR #9<br>#11 #3AND #10<br>#12 #11AND randomized controlled trial	371
Embase	#1 'Osteoporosis'/exp<br>#2 'Osteoporosis synonyms'/all fields<br>#3 #1 OR#2<br>#4 ‘Teriparatide’/exp<br>#5 ‘Teriparatide synonyms’/all fields<br>#6 #4 OR#5<br>#7 ‘Denosumab’/exp<br>#8 ‘Denosumab synonyms’/all fields<br>#9 #7 OR#8<br>#10 #6 OR #9<br>#11 #3AND #10<br>#12 #11AND randomized controlled trial	713
Cochrane Library	#1 Mesh descriptor: [Osteoporosis]<br>#2 (Osteoporosis synonyms): ti,ab,kw<br>#3 #1 OR#2<br>#4 Mesh descriptor: [Teriparatide]<br>#5 (Teriparatide synonyms): ti,ab,kw<br>#6 #4 OR#5<br>#7 Mesh descriptor: [Denosumab]<br>#8 (Denosumab synonyms): ti,ab,kw<br>#9 #7 OR#8<br>#10 #6 OR #9<br>#11 #3AND #10<br>#12 #11AND randomized controlled trial	1115
